# Transplant oncology and anti-cancer immunosuppressants

**DOI:** 10.3389/fimmu.2024.1520083

**Published:** 2025-01-07

**Authors:** Dejun Kong, Jinliang Duan, Shaofeng Chen, Zhenglu Wang, Jiashu Ren, Jianing Lu, Tao Chen, Zhuolun Song, Di Wu, Yuan Chang, Zhongqian Yin, Zhongyang Shen, Hong Zheng

**Affiliations:** ^1^ Nankai University School of Medicine, Tianjin, China; ^2^ Tianjin Organ Transplantation Research Center, Tianjin First Central Hospital, Nankai University School of Medicine, Tianjin, China; ^3^ Key Laboratory of Transplant Medicine, Chinese Academy of Medical Sciences, Tianjin, China; ^4^ Tianjin First Central Clinical College, Tianjin, China; ^5^ Research Institute of Transplant Medicine, Nankai University, Tianjin, China; ^6^ Tianjin Key Laboratory for Organ Transplantation, Tianjin, China

**Keywords:** organ transplantation, transplant oncology, immunosuppressant, anti-metabolic drugs, anti-tumor

## Abstract

Organ transplantation is a life-saving intervention that enhances the quality of life for patients with end-stage organ failure. However, long-term immunosuppressive therapy is required to prevent allogeneic graft rejection, which inadvertently elevates the risk of post-transplant malignancies, especially for liver transplant recipients with a prior history of liver cancer. In response, the emerging field of transplant oncology integrates principles from oncology and immunology to improve outcomes for patients at high risk of tumor occurrence or recurrence following transplantation. Therefore, it is of substantial clinical significance to develop immunosuppressants that possess both immunosuppressive and anti-tumor properties. For instance, mTOR inhibitors demonstrate anti-tumor effects among antimetabolite immunosuppressive drugs, and recent studies indicate that capecitabine, an antimetabolite chemotherapeutic, may also exhibit immunosuppressive activity in the clinic for liver transplants suffering from hepatocellular carcinoma. This review systematically explores potential immunosuppressants with dual anti-tumor and immunosuppressive effects to support the management of transplant patients at elevated risk of tumor occurrence or recurrence.

## 1 Introduction

Organ transplantation is the only way to extend the lives of many patients with end-stage organ failure. In 1963, Starzl et al. performed the first orthotopic liver transplantation, introducing the concept that primary liver malignancies unresponsive to conventional techniques, such as subtotal liver resection, should be considered for total hepatectomy and liver transplantation (1). Although transplant oncology is not a new concept, it has recently regained attention with advancements in transplant immunology and oncology, leading to the formal introduction of the term *Transplant Oncology* in 2015 (2). Transplant oncology involves replacing a cancerous organ with a healthy one and integrates multiple specialties from transplantation and oncology. It encompasses not only the surgical procedure but also the comprehensive management before and after organ transplantation. This multidisciplinary field relies on four pillars: the evolution of cancer care, the exploration of disease mechanisms, the elucidation of tumor and transplant immunology, and the extension of the limits of hepatobiliary cancer surgery. To date, liver transplantation remains the only solid organ transplantation that provides curative treatment for malignancies in selected patients (3).

However, after organ transplantation, cancer risk is one of the three major causes of death for the patients (4). Understanding of post-transplant malignancy is inadequate regarding early detection and lack of established guidelines. The risk factor is most elusive because of altered dynamics of immunity, host response, and different clinical presentation. Studies have shown an overall two to four-fold elevated risk of cancer after the organ transplant. The mechanisms involved in the oncogenesis are long-term immunosuppression leading to reduced immune surveillance of neoplastic cells, and the opportunistic post-transplant infections especially viral infections because of Epstein-Barr virus, Varicella, Cytomegalovirus and Human herpesvirus-8, etc. Physicians and patients face a challenging problem that cancer after an organ transplant is more biologically aggressive and patients may receive less aggressive cancer treatment because of comorbidities and the fear of transplant rejection.

Recurrence of hepatocellular carcinoma (HCC) following liver transplantation occurs in 6%–18% of patients transplanted for HCC (5). Recurrence is partially predictable and thus represents not only the loss of a potential donor resource, but also the added, and problematic challenge, of managing a presumably accelerated tumor progression in an immunocompromised host (6, 7). The Israel Penn International Transplant Tumor Registry, the largest and most comprehensive registry in the world records non-melanoma skin cancers as the most prevalent cancer in post organ transplant state. Following is the list of various cancers associated with the organ transplant: Kaposi sarcoma, skin (non-melanoma, non-epithelial), non-Hodgkin lymphoma, liver, anus, vulva, and lip tumors (8).

After organ transplantation, a triple therapy regimen, typically comprising calcineurin inhibitors (CNIs, such as tacrolimus or cyclosporine), mycophenolate mofetil (MMF), and glucocorticoids, is routinely administered for immunosuppression in the clinic (9, 10). However, experimental and early observational clinical studies have highlighted the interaction between immunosuppressive therapy and HCC recurrence (11). Several retrospective studies have demonstrated an association between CNIs levels and HCC recurrence. For example, one study found that recurrence was highest (46%) when high CNIs levels were present alongside other clinical or histological risk factors (Alpha-fetoprotein > 50ng/mL, macrovascular invasion, and G3–G4 grading). In patients with the same non-immunosuppressive risk factors but under low CNI levels, the recurrence rate was only 15%. These results link intensive immunosuppression with a high risk of tumor recurrence after liver transplantation (12). Therefore, for liver transplant patients with liver cancer or those at high risk of postoperative tumor development, it is necessary to introduce novel clinical strategies to prevent tumor recurrence after transplantation. Additionally, replacing CNIs with rapamycin drugs in the early postoperative period to mitigate the risk of tumor recurrence is also being adopted in clinical practice, this strategy decreases the rate of tumorigenesis after organ transplantation.

In 2004, Guba et al. proposed the notion that certain reagents with both anti-tumor and immunosuppressive effects could be used for liver transplantation in patients at high risk of tumor recurrence, we defined these agents referred to as anti-cancer immunosuppressants (ACIS) (13, 14). Additionally, they confirmed rapamycin as an effective ACIS in clinical settings. In recent years, some novel drugs have emerged as potential ACIS which may promote the development of transplant oncology, as shown in **Table 1**. In this review, we will summarize the current ACIS identified from experimental and clinical studies and mechanisms.

**Table 1:** Classification of immunosuppressants with anti-tumor effects.

Classification	The name of the drug	Clinical functions	Reference
Mammalian target of rapamycin inhibitors and derivates	Sirolimus	Prevent allograft rejection	(25)
	Everolimus	Prevent allograft rejection	(24)
Antimetabolites	5-Fluorouracil and Capecitabine	Cancer treatment	(26–37)
	Mycophenolic acid and its derivatives	Prevent allograft rejection	(38–45)
	Cyclophosphamide	Cancer treatment Immunomodulation	(46–51)
	6-mercaptopurine and its derivatives	Cancer treatment	(52–55)
	Methotrexate	Immunomodulation	(56–60)
	L-asparaginase	Cancer treatment	(61–65)
Traditional Chinese herb-derived drugs	Icaritin	Cancer treatment	(66–79)
	Artemisinin and Its Derivatives	Anti-malarial	(80–101)
	Berberine	Anti-inflammatory	(102–113)
	Paclitaxel	Cancer treatment	(114–119)
	Oxymatrine	Antiviral	(120–128)
	Fingolimod Hydrochloride	Immunomodulation	(129–132)
	Luteolin	Anti-inflammatory	(133, 134)
	Shikonin	Antiviral	(135, 136)
	Emodin	Antidepressant	(137, 138)

## 2 Mammalian target of rapamycin inhibitors and derivates

**Figure 1:**
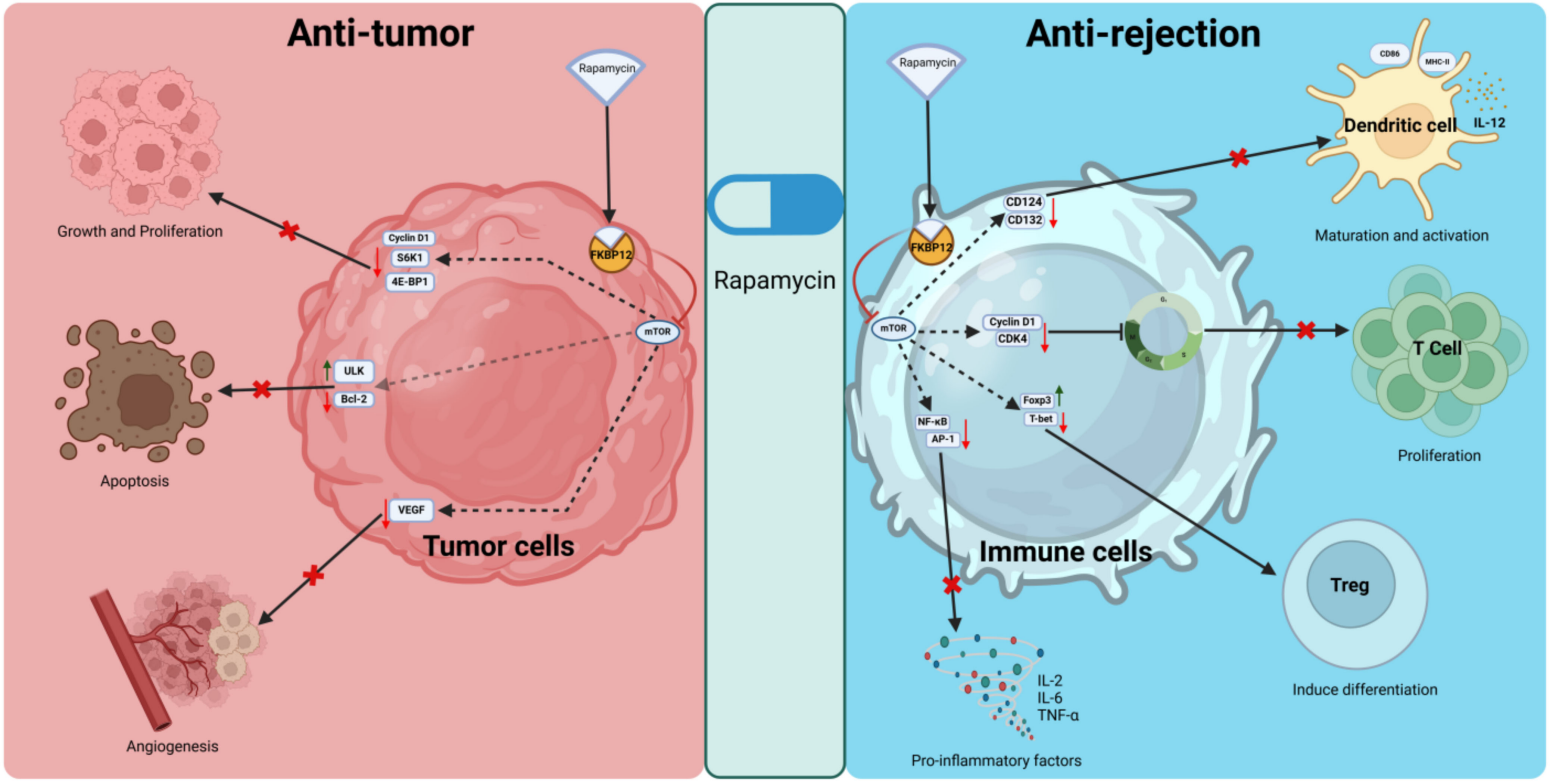
Anti-tumor and anti-rejection mechanisms of rapamycin. Rapamycin binds to FKBP12, forming a complex that inhibits mTOR. In tumor cells, it suppresses proliferation by downregulating cyclin D1, S6K1, and 4E-BP1, promotes apoptosis by regulating ULK and Bcl-2, and inhibits angiogenesis by reducing VEGF levels. In immune cells, it inhibits dendritic cell maturation, T cell proliferation, and pro-inflammatory cytokine production while promoting regulatory T cell induction through modulation of key signaling pathways and transcription factors. S6K1, ribosomal protein S6 kinase 1; 4E-BP1, eukaryotic translation initiation factor 4E-binding protein 1; ULK, Unc-51 like autophagy activating kinase; Bcl-2, B-cell lymphoma 2; CD124, interleukin-4 receptor subunit alpha; CD132, interleukin-2 receptor subunit gamma; CDK4, cyclin-dependent kinase 4.

Interestingly, immunosuppression is correlated with tumor occurrence risk (15), some HCC pathways are also the target of some immunosuppression agents, such as the mammalian target of rapamycin (mTOR) inhibitors (mTORi) (16, 17) (**Figure 1**). Indeed, activation of the PI3K/AKT/mTOR pathway is common (50%–60%) in HCC, and is correlated with poor HCC prognosis. Retrospective studies have documented lower HCC recurrence and higher posttransplant survival in patients under mTORi immunosuppression compared to CNIs. In the largest study (n = 2491 HCC patients and 12167 non-HCC patients), sirolimus (SRL)-based immunosuppression was associated with improved 5-year survival in those with an HCC indication (18, 19).

In HCC, mTOR promotes protein synthesis by activating S6K1 and inhibiting 4E-BP1, both of which enhance protein translation. This supports the uncontrolled growth and proliferation of HCC cells. mTOR controls glucose metabolism, lipid synthesis, and mitochondrial function (20). In HCC, hyperactivation of mTOR drives anabolic processes, providing the energy and biomass needed for rapid tumor growth. mTOR also regulates angiogenesis through vascular endothelial growth factor (VEGF) production, ensuring an adequate blood supply for the expanding tumor mass in HCC. Under normal conditions, mTOR inhibits autophagy, a cellular degradation process. In HCC, sustained mTOR activation suppresses autophagy, which may contribute to tumor survival by preventing the clearance of damaged organelles and proteins (17, 21, 22).

### 2.1 Sirolimus and Everolimus

While in transplantation, rapamycin binds to *FKBP12* and inhibits mTOR, which blocks T-cell and B-cell activation and proliferation by preventing the response to IL-2 (23). SRL and Everolimus are both mTOR inhibitors that share a similar mechanism of action but differ in their chemical properties, pharmacokinetics, and clinical applications (24, 25). Both SRL and Everolimus are used as immunosuppressants to prevent organ rejection after transplantation, but they have different pharmacokinetic profiles and clinical applications. 

## 3 Antimetabolites

### 3.1 5-Fluorouracil and Capecitabine

5-Fluorouracil (5-FU) is a uracil analogue wherein the hydrogen at the 5-position of uracil is substituted with fluorine. As a quintessential antimetabolic agent, it has been extensively utilized in clinical oncology for the treatment of various malignancies. 5-FU itself lacks inherent biological activity and must be metabolized *in vivo* to its active forms: fluorouracil deoxynucleotide (F-dUMP) and fluorouracil triphosphate nucleoside (FUTP). F-dUMP, structurally analogous to dUMP, inhibits deoxythymidine synthase, while FUTP is integrated into RNA as FUMP. In addition to its antitumor properties, 5-FU exhibits significant immunosuppressive effects, which may facilitate its use in preventing organ rejection post-transplantation. Prior research has demonstrated that 5-FU can extend the survival of rat heart allografts (26). Nonetheless, the potential for serious side effects, such as bone marrow suppression, constrains the feasibility of its prolonged use in transplant background.

Capecitabine (CAP), a prodrug of 5-FU, has emerged as a first-line chemotherapeutic agent for the treatment of malignancies such as colorectal cancer. Following oral administration, CAP is absorbed in the gastrointestinal tract and subsequently metabolized into its intermediary forms: 5’-deoxy-5-fluorocytidine and 5’-deoxy-5-fluorouridine, through the action of carboxylesterase and cytidine deaminase, respectively. Ultimately, these intermediates are converted to 5-FU by thymidylate phosphorylase (TP). The expression of TP is markedly elevated in cancerous tissues compared to adjacent normal tissues, leading to a higher accumulation of 5-FU in tumor cells and enhanced anticancer efficacy (27). Consequently, the therapeutic effectiveness of CAP is closely associated with the distribution and expression of TP.

Early research has established that CAP is effective in preventing acute rejection of canine renal allografts. Although neurotoxicity has been observed, it can be attenuated by dose reduction (28, 29). Subsequent studies have demonstrated that, in addition to its presence in tumor cells, TP is highly expressed in T cells, whereas its expression in bone marrow tissue remains minimal. This differential tissue distribution of TP not only mitigates the myelosuppressive side effects of CAP but also promotes selective enrichment of 5-FU in T cells, thereby minimizing its cytotoxic effects. Research has demonstrated that CAP reduces the proportion of T cells in mice and lowers the levels of associated proinflammatory factors, including IL-2, TNF-α, and IL-6, suggesting that CAP primarily exerts its immunosuppressive effects on compromised T cells (30). Further investigations revealed that CAP can T cell apoptosis by inhibiting the AKT/SMARCC1/AP-1 axis through HSP90AB1 (31). Metronomic chemotherapy is characterized by the frequent administration of conventional chemotherapy drugs at low doses with no prolonged drug-free breaks. Compared to traditional chemotherapy, it not only exerts direct effects on tumor cells, but also inhibits angiogenesis by inhibiting the synthesis and release of anti-angiogenic factors inducing thrombospondin-1, endostatin, angiostatin and pigment epithelium-derived factor (32). In addition, it minimizes the cytotoxicity of conventional chemotherapy drugs (33). In a clinical study examining liver cancer recurrence following liver transplantation, metronomic CAP achieved comparable efficacy to sorafenib without causing acute rejection, indicating its potential as an anti-rejection therapy post-transplantation (34). Zheng et al. has shown that metronomic CAP administered to recipient rats can induce T cell ferroptosis by inhibiting the Nrf2/HO-1/GPX4 antioxidant system, thereby mitigating liver allograft rejection (35). Additionally, they demonstrated that CAP targets thymidylate synthase (TYMS) inhibition, an enzyme essential for DNA synthesis and pyrimidine metabolism, thereby suppressing CD4+ T cell activation and Th1 cell differentiation, effects that contribute to CAP-mediated inhibition of cardiac allograft rejection (36). Further studies have confirmed that rapamycin enhances the protective effects of metronomic CAP, prolonging survival in rat liver transplantation and murine cardiac allograft models (37). At present, the immunosuppressive effects of metronomic CAP on organ transplantation have only been demonstrated in experimental animal models, highlighting the need for further basic and clinical research to validate these findings.

### 3.2 Mycophenolic acid and its derivatives

Mycophenolic acid (MPA), an antibiotic derived from Penicillium stoloniferum and similar fungal strains, inhibits the proliferation and function of T and B lymphocytes by targeting inosine monophosphate dehydrogenase, the rate-limiting enzyme in the *de novo* synthesis of guanine nucleotides. Currently, MPA and its derivatives, including MMF and mycophenolate sodium, are predominantly employed clinically as immunosuppressants in organ transplantation and for the treatment of autoimmune diseases such as systemic lupus erythematosus and rheumatoid arthritis.

Studies have substantiated the anti-tumor effects of MPA and its derivatives both *in vivo* and *in vitro*. Previous research indicates that MPA effectively inhibits the proliferation of various human and mouse tumor cell lines in a dose-dependent manner, ranging from low doses (0.01 μM) to high doses (100 μM) *in vitro*. Tumor cell lines exhibit varying degrees of sensitivity to MPA, with human lymphocytic leukemia cells (Molt-4) demonstrating the highest susceptibility (IC50 < 0.1 μM). Conversely, not all tumor cell lines are responsive to MPA; for instance, gastric adenocarcinoma cells (Hs746T), pancreatic ductal carcinoma cells (PANC-1), and human HCC cells (HepG2) show resistance to MPA’s antiproliferative effects, even after 72 hours of treatment with concentrations up to 20 μg/mL (38). In alignment with these *in vitro* findings, numerous studies have reported that MMF, when administered either by gavage or intraperitoneal injection, can inhibit the growth and metastasis of several tumors in tumor-bearing mice or rats. However, certain tumors, such as mouse colon adenocarcinoma cells (CT26), liver endothelioma cells (SK-Hep-1), and liver adenocarcinoma cells (Hep-3B), exhibit resistance to MMF when implanted in animal models (39, 40).

Clinical studies have demonstrated that the inclusion of MMF in standard immunosuppressive regimens is associated with a significantly reduced risk of developing malignancies (41–43). Two notable clinical trials have investigated the anti-cancer effects of MMF. In 2004, Takebe et al. conducted a Phase I clinical trial to evaluate MMF for the treatment of advanced multiple myeloma. The study revealed that patients with relapsed and refractory multiple myeloma tolerated MMF well at doses ranging from 1 to 5 grams per day, and the clinical response correlated positively with a decrease in intracellular deoxyguanosine triphosphate levels (44). In another trial, 12 patients with resectable pancreatic ductal adenocarcinoma were treated with MMF or a placebo for 5 to 15 days prior to surgery. Unfortunately, the MMF-treated group (n = 6) did not exhibit significant anti-tumor effects compared to the untreated group (n = 6) (45). Consequently, further investigation is required to fully understand the therapeutic potential of MPA-based drugs across various malignancies.

### 3.3 Cyclophosphamide

Cyclophosphamide (CP) is a nitrogen mustard derivative that, upon hydrolysis by phosphoramidase or phosphatase in the liver or tumor tissue, is converted into its active form, phosphoramide mustard. This active metabolite induces cross-linking of DNA by alkylating guanine bases, leading to the formation of cross-links between DNA strands or within the same strand, thereby disrupting the DNA structure. As a cytotoxic chemotherapeutic agent, CP is utilized clinically for the treatment of various malignancies, including malignant lymphoma, multiple myeloma, breast cancer, small cell lung cancer, and ovarian cancer. Additionally, CP serves as an immunosuppressant in the management of autoimmune diseases and in preventing organ rejection during transplantation.

In 1963, Berenbaum first demonstrated that a single dose of CP could extend the survival of skin allografts in mice (46). In 1971, Starzl et al. reported the application of CP in human organ transplantation (47). Following this, CP was employed clinically to prevent rejection in kidney, heart, liver, and other organ transplants (48–50). However, as an early immunosuppressive agent, due to its significant toxicities, including bladder toxicity and gonadotoxicity, CP has been increasingly supplanted by safer alternatives, such as cyclosporine A (51).

### 3.4 6-mercaptopurine and its derivatives

6-mercaptopurine (6-MP) is a purine analog that impedes cell proliferation and induces cell death by interfering with DNA and RNA synthesis. It is primarily utilized in the clinical management of leukemia. Given that activated and proliferating lymphocytes involved in rejection are heavily reliant on purine metabolism, 6-MP can function as an immunosuppressive agent in organ transplantation. In 1960, Zukoski demonstrated that 6-MP could extend the functional survival of renal homografts in canines (52).

Azathioprine (AZA) is a sustained-release prodrug of 6-MP. Although AZA and 6-MP exhibit comparable immunosuppressive activities *in vivo*, AZA is associated with reduced toxicity at equivalent immunosuppressive concentrations. Consequently, AZA is extensively utilized in the treatment of autoimmune diseases and in the immunosuppressive management of organ transplantation (53). The first application of AZA for immunosuppression in human kidney transplantation occurred in 1961 (54). Subsequently, AZA, in combination with corticosteroids and CNIs, became the standard regimen for immunosuppressive drug therapy in organ transplantation (55). However, in recent years, AZA has been progressively supplanted by MMF, another purine nucleotide inhibitor, owing to MMF’s superior immunosuppressive efficacy compared to AZA (55).

### 3.5 Methotrexate

Methotrexate (MTX) is a folate analog that inhibits dihydrofolate reductase, thereby preventing the reduction of dihydrofolate to the physiologically active tetrahydrofolate. This inhibition disrupts the transfer of one carbon units during the biosynthesis of purine and pyrimidine nucleotides, ultimately leading to the inhibition of DNA synthesis. Additionally, MTX exerts an inhibitory effect on TYMS; however, its effects on RNA and protein synthesis are relatively weak. Initially developed over 70 years ago as an anti-folate chemotherapy agent, MTX is now widely utilized as a first-line treatment for autoimmune and inflammatory diseases such as rheumatoid arthritis, psoriasis, and Crohn’s disease, etc. (56).

Alexandra et al. demonstrated that the combination of ATG and MTX can achieve long-term survival of mouse heart allografts through the induction of immune tolerance (57). In a clinical study, MTX was utilized as a rescue or adjuvant therapy in both adult and infant heart transplant recipients. The results indicated that while one case of grade 3b rejection was not reversed by MTX, the rejections in all other cases were swiftly reversed following MTX treatment (58). Subsequently, another study investigating the use of MTX in conjunction with conventional triple immunosuppressive therapy for recurrent mild to moderate acute rejection in pediatric heart transplantation found that the frequency of rejection at two months post-MTX treatment was significantly reduced compared to pre-treatment levels (59). However, an additional clinical study on heart transplantation revealed that patients treated with MTX experienced a higher rejection rate after MTX reversal than those who did not receive MTX (60). Therefore, the potential long-term benefits of MTX for organ transplant recipients warrant further investigation.

### 3.6 L-asparaginase

L-asparaginase, an amidohydrolase, is the first enzyme used in cancer treatment and is widely found in various animals, plants, and microorganisms, including bacteria, fungi, algae, yeast, and actinomycetes, but not in humans (61). Clinically, L-asparaginase is primarily extracted and isolated from Escherichia coli bacteria for the treatment of acute lymphoblastic leukemia. This anti-tumor drug exerts cytotoxic effects by inhibiting intracellular protein synthesis. Specifically, L-asparaginase catalyzes the hydrolysis of L-asparagine into L-aspartate and ammonia in the bloodstream. Normal cells, equipped with asparagine synthetase, can synthesize L-asparagine, whereas leukemia cells, deficient in asparagine synthetase, are unable to synthesize sufficient L-asparagine, leading to impaired protein synthesis and subsequent anti-tumor effects (62).

Early studies have established the immunosuppressive effects of L-asparaginase. Following treatment with L-asparaginase, mice exhibited reduced lymphocyte counts, atrophy of the lymph nodes, thymus, and spleen, and impaired migration of lymph node cells to the lymph nodes when injected into recipients of the same genotype. *In vivo* experiments have further demonstrated that L-asparaginase can mitigate skin graft rejection and inhibit the production of antibodies against sheep red blood cells (63). Additionally, Rapaport et al. confirmed that L-asparaginase can prolong the survival of canine renal allografts (64), and Levin et al. verified its immunosuppressive effects in an inbred rat renal transplant model (65). However, there are few reports on the immunosuppressive effects of L-asparaginase and its application in clinical solid organ transplantation.

## 4 Traditional Chinese herb-derived drugs

Traditional Chinese herbs or plant extracts have demonstrated promising antitumor effects in preclinical studies, while also showing immunosuppressive properties in organ transplantation models, suggesting their potential as novel immunosuppressants with antitumor effects.

### 4.1 Icaritin

Icaritin (ICT) is an active monomer extracted and isolated from the epimedium plant. Due to its potent therapeutic effects against human malignancies, ICT has entered clinical trials for the treatment of PD-L1-positive advanced HCC (Phase III, ClinicalTrials, NCT03236649), HCC (Phase III, ClinicalTrials, NCT03236636), advanced solid tumors (Phase Ib, completed, ClinicalTrials, NCT02496949), and advanced breast cancer (Phase I, ClinicalTrials, NCT01278810). The antitumor mechanisms of ICT include the downregulation of cyclin-dependent kinase 2 (CDK2) (66–68), inducing cell cycle arrest in HCC, colon cancer, and prostate cancer. ICT activates NF-related apoptosis-inducing ligand, Fas-caspase-3/8 (69, 70), inhibits the phosphorylation of Akt and mTOR (71, 72), activates PTEN/Parkin-dependent mitochondrial autophagy (73), downregulates Jak-2, p-Stat3, and p-Akt expression (74), and upregulates p-JNK and p-C-Jun expression (75, 76), thereby inducing apoptosis in human glioblastoma, liver cancer, ovarian cancer, and chronic myeloid leukemia cells. Additionally, ICT suppresses tumor cell glycolysis by upregulating *p53*, thereby inhibiting energy metabolism and hindering liver cancer growth (77). ICT also modulates the tumor immune microenvironment by directly binding to MyD88 or IKKα to inhibit the TLR-MyD88-IKK-NF-κB inflammatory pathway, reducing the production of inflammatory cytokines such as TNF-α and IL-6 (78). In organ transplantation, ICT has been reported to extend the survival time of allogeneic skin grafts by inhibiting T cell activation (79), indicating its immunosuppressive effects. While ICT is already in clinical use as an antitumor drug, its immunosuppressive effects in organ transplantation warrant further investigation.

### 4.2 Artemisinin and its derivatives

Artemisinin (ART) and its derivatives are natural compounds extracted from the artemisia annua plant, a member of the Asteraceae family, which has been traditionally used in Chinese medicine. They are renowned for their potent antimalarial properties. In addition to their antimalarial effects, ART and its derivatives have also demonstrated significant antitumor activity. Furthermore, in allogeneic transplant animal models, ART and its derivatives have notably extended graft survival, suggesting potential immunosuppressive effects.

#### 4.2.1 Artemisinin

The antitumor effects of ART began to receive attention in the early 1990s (80). ART induces cell cycle arrest in human prostate cancer, human gallbladder cancer, and human breast cancer cells by downregulating the expression of cyclin-dependent kinases CDK2 and CDK4 (81–83). ART reduces the expression of p-ERK1/2 and inhibits the proliferation of human gallbladder cancer cells (82). Besides its antimalarial and antitumor properties, ART has also been reported to possess immunosuppressive effects. In rat models of allogeneic cardiac transplantation, oral administration of ART reduced the infiltration of effector T cells and the secretion of inflammatory cytokines, increased the infiltration of regulatory T cells, and decreased macrophage infiltration in the graft, thereby significantly extending graft survival (84). Additionally, ART significantly prolonged the survival time of mouse allogeneic skin grafts, which is associated with the blockade of the OX40-OX40L co-stimulatory signaling pathway and reduced IL-6 secretion (85).

#### 4.2.2 Artesunate

Artesunate (ARS) is derived from the esterification of dihydroartemisinin and succinic anhydride, and it exhibits better aqueous solubility compared to ART. Multiple clinical studies have shown that ARS treatment results in a significant inhibition of tumor growth (86–90). ARS affects signaling pathways and transcription factors associated with tumor growth, including the inhibition of the IL-6-JAK-STAT pathway (91), reduction of NF-κB p65 transcriptional activity (92), activation of p38 MAPK (93), and suppression of the Wnt/β-catenin pathway (94). ARS inhibits the proliferation of rat HCC, mouse multiple myeloma, human acute myeloid leukemia, and rhabdomyosarcoma cells. ARS also suppresses the secretion of VEGF and its receptor KDR/flk-1, and inhibits microvascular formation in human ovarian cancer (95). By upregulating intracellular free iron levels, ARS promotes the accumulation of intracellular lipid peroxides and induces ferroptosis in human rhabdomyosarcoma, Burkitt’s lymphoma, human pancreatic cancer, human cervical cancer, and human fibrosarcoma cells (93, 96–99). ARS induces oxidative DNA damage leading to DNA double-strand breaks in human glioblastoma cells (100). ARS has also been reported to have immunosuppressive effects. In mouse allogeneic cardiac transplantation models, ARS significantly reduced the infiltration of inflammatory cells in cardiac tissue and the proportion of CD4+ CD8+ T cells in the spleen and lymph nodes, decreased cardiomyocyte apoptosis, and extended graft survival by reducing the expression of PERK, ATF4, and CHOP to mitigate ROS production and endoplasmic reticulum stress, and by inhibiting dendritic cell maturation (101).

### 4.3 Berberine

Berberine (BBR) is an isoquinoline alkaloid extracted from Coptis chinensis and Phellodendron amurense, commonly used in the treatment of bacterial diarrhea (102). Several preclinical studies have demonstrated BBR’s antitumor properties. BBR induces cell cycle arrest in human thyroid cancer and glioblastoma cells by upregulating the expression of CIP1/p21 and Kip1/p27 proteins and downregulating cyclin-dependent kinases (CDK2, CDK4, CDK6) (103, 104). It inhibits the activation of transcription factor AP-1 (105), blocks the nuclear translocation of p50/p65 NF-κB proteins and their binding to the COX-2 promoter (106), thereby suppressing the proliferation of human cervical cancer and non-small cell lung cancer cells. BBR induces apoptosis in human liver cancer cells by upregulating *P53* (107), downregulating CD147 (108), increasing Bax expression, activating caspases 3 and 9 (109), promoting AMPK phosphorylation, and inhibiting Akt phosphorylation (110). Additionally, BBR inhibits the expression of MMP-2 (111), suppresses the expression of Hypoxia-inducible factor-1α, Vascular endothelial growth factor, and Pigment epithelium-derived factor (106), and interacts with vasodilator-stimulated phosphoprotein (112), thereby reducing microvascular proliferation and migration in human non-small cell lung cancer and breast cancer cells. In animal models of solid organ transplantation, BBR has shown immunosuppressive effects. In a murine cardiac allograft model, BBR treatment via intraperitoneal injection significantly extended graft survival through inhibiting the activation and proliferation of CD4+ and CD8+ T cells and inducing T cell apoptosis through activation of the mitochondrial apoptosis pathway (e.g., inducing expression of PCNA, Bcl-2, Bax, cytochrome c, cleaved-caspase-3, and cleaved-PARP), thus prolonging the survival of cardiac allografts in mice (113). Due to its low water solubility and poor oral absorption, BBR has not yet been applied clinically.

### 4.4 Paclitaxel

Paclitaxel (PTX) is a diterpenoid alkaloid extracted from the Pacific yew tree, known for its anti-cancer properties (114). It has been approved by the U.S. Food and Drug Administration for the treatment of various cancers, including breast cancer, non-small cell lung cancer, and ovarian cancer. PTX exerts its effects by binding to the β-tubulin subunit of microtubules, preventing their depolymerization, thereby stabilizing the microtubule structure. This stabilization inhibits tumor cell proliferation, migration, and neovascularization, while promoting apoptosis in tumor cells (115, 116). The microtubule stabilization induced by PTX is associated with the phosphorylation of Bcl-2, which prevents its interaction with calcineurin and subsequent chelation, thereby allowing the activation of T-cell nuclear factor and subsequent transcription of CD95L (117). This is related to alloantigen-induced lymphocyte activation, suggesting a potential immunosuppressive role for PTX. Studies have shown that intraperitoneal administration of PTX significantly prolongs the survival of allografts in rat heart transplantation models. PTX induces lymphocyte apoptosis, reducing the activity of cytotoxic T cells and the production of IL-2, as well as the levels of cytotoxic antibodies after alloantigen re-exposure (118, 119).

### 4.5 Oxymatrine

Oxymatrine (OMT) is an alkaloid compound extracted from the traditional Chinese medicine *sophora flavescens*. OMT is utilized for the treatment of chronic hepatitis, chronic bronchitis, and other conditions. Preclinical studies have demonstrated its anti-tumor effects. OMT reduces the expression of Cyclin D1, CDK4, and CDK6 while enhancing P21 expression (120, 121), leading to cell cycle arrest in human esophageal and gastric cancer cells. It upregulates p53 and Bax proteins (122), increases caspase-3/caspase-9 activity (123), and inhibits the expression of PI3K, p-Akt, p-mTOR, p-p70S6K, and NF-κB (124), inducing apoptosis in human gallbladder, prostate cancer, and acute leukemia cells. In addition, OMT decreases the expression levels of VEGF (125), TGF-β1, and PAI-1 proteins (126), and inhibits tumor micro-vessel formation, invasion, and metastatic potential in human pancreatic and colorectal cancers. In a murine cardiac allograft model, intraperitoneal administration of OMT significantly prolonged graft survival reduced CD3+ T cell infiltration, increased Treg cell infiltration, and suppressed the capacity of splenic dendritic cells to stimulate T cell proliferation, alleviating acute graft rejection (127). The combination of OMT with rapamycin was more effective than either treatment alone in inhibiting mTOR and HIF-1α expression in CD4+ or CD11c+ cells within the graft (128).

### 4.6 Fingolimod hydrochloride

FTY720, a sphingosine-1-phosphate (S1P) analog derived from the ascomycete Cordyceps sinensis is a potent immunosuppressant widely used in clinical settings for the treatment of multiple sclerosis (129). After phosphorylation, FTY720 binds to S1P receptors to exert its immunosuppressive effects. The non-phosphorylated form of FTY720 induces apoptosis, enhances chemotherapy sensitivity, and inhibits tumor metastasis by suppressing sphingosine kinase 1 and activating protein phosphatase 2A along with various cell death pathways (130). In the field of organ transplantation, Chiba first demonstrated in 1996 that FTY720 could extend the survival of skin, heart, and kidney allografts in rats and dogs (131). Subsequent studies have validated FTY720’s anti-rejection effects in other organ transplantation models, including liver transplants in dogs, and explored its combination with other immunosuppressive agents (132). Additionally, other herbal extracts such as Luteolin (133, 134), Shikonin (135, 136) and Emodin (137, 138) have been reported to exhibit both anti-tumor and immunosuppressive effects.

Although these herbal extracts have demonstrated promising effects in preclinical studies, the absence of relevant Phase III clinical trials highlights the challenges in clinical translation. Key issues requiring further investigation include the standardization and quality control of the extracts, the analysis of their absorption, metabolism, and excretion in the human body, and the monitoring of potential adverse drug reactions.

## 5 Conclusion and perspectives

In this review, we comprehensively address CAP, an antimetabolic drug, as a promising candidate for ACIS. Evidence suggests that metronomic CAP treatment, an emerging anti-tumor strategy for liver transplant patients with recurrent tumors, can effectively prevent allograft rejection while simultaneously delaying tumor progression (34). Metronomic chemotherapy is clinically advantageous due to its low side effects and suitability for long-term oral administration, offering substantial benefits to patients (139–142). Given that drug monotherapy alone is often insufficient to halt tumor progression or prevent graft rejection in clinical practice, combination therapies are frequently employed. Therefore, we propose criteria for identifying potential ACIS agents for clinical use: agents should effectively inhibit both tumor progression and allograft rejection, be suitable for oral and long-term administration, and demonstrate compatibility with other immunosuppressants to enhance therapeutic efficacy.

In addition, it is worth noting that the use of immune checkpoint inhibitors (ICIs) in solid organ transplant recipients as an adjuvant therapy for recurrent HCC may cause a severe alloimmune injury, even when the drug was introduced years after transplantation (143). In addition, recipients who discontinue ICIs days before transplantation also face a high risk of rejection (144, 145). A safe period between pretransplant ICI administration and transplant remains to be determined. Therefore, ICIs should be used with extreme caution in the peri-transplant or post-transplant.

Allograft rejection and tumor occurrence are two main factors that hinder the long-term survival of transplants, proposing a novel strategy to prevent both rejection and tumor will benefit the patients. Overall, a comprehensive exploration of ACIS warrants further investigations, and we hope that our review and perspectives will contribute to advancements in the field of transplant oncology.

## Glossary

HCC: Hepatocellular Carcinoma
IPITTR: Israel Penn International Transplant Tumor Registry
NMSCs: Non-melanoma Skin Cancers
CNIs: Calcineurin Inhibitors
MMF: Mycophenolate Mofetil
ACIS: Anti-tumor and Immunosuppressant Compounds
mTOR: mammalian target of rapamycin
PI3K: Phosphatidylinositol-3-kinase
AKT: Akt serine/threonine kinase
SRL: Sirolimus
IL-2: Interleukin-2
5-FU: 5-Fluorouracil
F-dUMP: Fluorouracil Deoxynucleotide
FUTP: Fluorouracil Triphosphate Nucleoside
CAP: Capecitabine
TP: Thymidylate Phosphorylase
TNF-α: Tumor Necrosis Factor-alpha
IL-6: Interleukin-6
SMARCC1: SWI/SNF Related, Matrix Associated, Actin Dependent Regulator of Chromatin, Subfamily C, Member 1
AP-1: Activator Protein 1
HSP90AB1: Heat Shock Protein 90 Alpha Family Class B Member 1
Nrf2: Nuclear Factor Erythroid 2-related Factor 2
HO-1: Hemoxygenase-1
GPX4: Glutathione Peroxidase 4
TYMS: Thymidylate Synthase
MPA: Mycophenolic Acid
CP: Cyclophosphamide
6-MP: 6-mercaptopurine
AZA: Azathioprine
MTX: Methotrexate
ICT: Icaritin
PD-L1: Programmed Cell Death Ligand 1
CDK2: Cyclin-Dependent Dinase 2
Jak-2: Janus kinase 2
p-Stat3: phosphorylated Signal Transducer and Activator of Transcription 3
p-Akt: phosphorylated Akt serine/threonine kinase
p-JNK: phosphorylated c-Jun N-terminal Kinase
p-C-Jun: phosphorylated c-Jun
MyD88: Myeloid Differentiation Primary Response 88
IKKα: IκB Kinase α
TLR: Toll-Like Receptor
NF-κB: Nuclear Factor kappa-light-chain-enhancer of activated B cells
ART: Artemisinin
CDK4: Cyclin-Dependent Dinase 4;p-ERK1/2, phosphorylated Extracellular Signal-Regulated Kinase 1/2
ARS: Artesunate
MAPK: Mitogen-Activated Protein Kinase
VEGF: Vascular Endothelial Growth Factor
KDR: Kinease Domain Receptor
flk-1: Fetal Liver Kinase 1
PERK: Protein Kinase RNA-like Endoplasmic Reticulum Kinase
ATF4: Activating Transcription Factor 4
CHOP: C/EBP Homologous Protein
ROS: Reactive Oxygen Species
BBR: Berberine
CIP1/p21: Cyclin-dependent kinase inhibitor 1
Kip1/p27: Cyclin-dependent kinase inhibitor 1B
COX-2: Cyclooxygenase-2
MMP-2: Matrix Metalloproteinase-2
PCNA: Proliferating Cell Nuclear Antigen
Bcl-2: B-cell Lymphoma 2
Bax: Bcl-2 Associated X Protein
PTX: Paclitaxel
OMT: Oxymatrine
p-p70S6K: phospho-p70 S6 Kinase
TGF-β1: Transforming Growth Factor Beta 1
PAI-1: Plasminogen Activator Inhibitor-1
S1P: Sphingosine-1-Phosphate.
